# Physical Activity and Sedentary Behaviour in Children With Haemophilia

**DOI:** 10.1111/hae.70347

**Published:** 2026-07-03

**Authors:** Melanie Bladen, Lucy Alderson, Eleanor Main, Mario Cortina‐Borja

**Affiliations:** ^1^ Great Ormond Street Hospital for Children NHS Foundation Trust London UK; ^2^ Physiotherapy UCL Great Ormond Street Institute of Child Health London UK; ^3^ Population, Policy and Practice Research and Teaching Department UCL Great Ormond Street Institute of Child Health London UK

**Keywords:** accelerometer, children, haemophilia, physical activity, sedentary behaviours

## Abstract

**Introduction:**

Physical activity (PA) is vital in child development, while inactivity remains a leading contributor to global mortality. Therapeutic advances have enabled greater PA in boys with haemophilia (BwH), yet robust UK data on objectively measured PA and sedentary behaviour (SB) across severities are limited.

**Aim:**

To investigate PA and SB in BwH and assess adherence to World Health Organization (WHO) guidelines for moderate‐to‐vigorous physical activity (MVPA).

**Methods:**

Sub‐analysis of a prospective cohort at a UK haemophilia centre (2018). Participants (*n* = 36; 6–16 years; all severities including inhibitors) wore hip mounted ActiGraph GT3X accelerometers for 7 days. Valid data required ≥ 9 h/day for ≥ 4 days. MVPA and SB were analysed using Evenson thresholds. Mixed‐effects models with random intercepts examined demographic/clinical correlates of MVPA and SB and the odds of achieving >60 min/day of MVPA.

**Results:**

Across 216 valid days median daily MVPA was 52.5 min/day (IQR: 30.9–80.2), with no significant differences by disease severity. Age was the only significant predictor of MVPA and SB: each additional year was associated with 7.6 fewer minutes of MVPA and 27.6 more minutes of SB per day (*p* < 0.01). Only 39.8% of valid days met the WHO MVPA guidelines. The probability of meeting this target declined from 79% at age 6to 8% at age 14.

**Conclusions:**

Most BwH did not meet WHO MVPA guidelines, though activity levels were encouraging. Age‐related declines in MVPA and increases in SB mirror those seen in the general population, underscoring the need for early, tailored interventions to support lifelong activity in haemophilia.

## 1 Introduction

Physical activity (PA) contributes to childhood quality of life, impacting future physical, psychological and social development and well‐being [1]. Physical inactivity or sedentary behaviour (SB) is a major global risk factor for mortality and contributes significantly to increasing rates of overweight, obesity and non‐communicable diseases [2]. Although SB is a natural part of the human movement spectrum, it can adversely affect health, and is influenced by, but not limited to, social, environmental, economic and technological factors [3]. SB is widespread in modern society, with much of the population exposed to the health risks associated with prolonged sitting. Extended periods of inactivity can negatively impact health, including reduced cardiorespiratory fitness and changes in muscle fibre composition [3]. Early childhood is a critical period of rapid physical and cognitive development, during which PA and SB patterns are malleable and can be shaped for long‐term health [4]. These established patterns also provide a valuable foundation for examining PA and SB in children with haemophilia.

Advances in medical care and prophylaxis have reduced bleeding episodes in boys with haemophilia (BwH), enabling greater participation in PA [5]. PA offers multiple benefits for BwH including reduced bleeding frequency, improved muscle strength, proprioception, balance and bone mineral density. Adequate activity, particularly weight‐bearing exercises, is one of the main determinants for achieving peak bone mass. Adolescence represents a critical and challenging period for this development [6]. The World Federation of Haemophilia supports PA for BwH, highlighting its role in enhancing quality of life [7]. PA should be tailored to each child's age, needs, abilities and interests to promote physical fitness and neuromuscular development [7]. Most BwH on prophylaxis can now lead active lives and are encouraged to follow World Health Organization (WHO) guidelines for PA: an average of 60 min/day of moderate‐to‐vigorous PA (MVPA) across the week, while limiting sedentary time, particularly recreational screen use [4].

While advances in haemophilia care have improved life expectancy for people with haemophilia (PwH), studies show higher rates of hypertension, insulin resistance and hyperlipidaemia in this group compared to controls [8]. Low PA levels, reduced physical fitness, abdominal adiposity and hypertension may increase the risk and severity of cardiometabolic and musculoskeletal health conditions as PwH age [8]. Establishing healthy PA, reduced SB, and nutritious eating habits early in life, help shape long‐term behaviours into adulthood [9, 10]. Education, health promotion, physical literacy, PA and reducing SB are key strategies for addressing risk factors linked to cardiometabolic disease in adulthood.

While self‐reported measures of physical function in PwH are common in the literature [11], activity trackers provide more accurate, objective data. However, methodological and practical differences in wearable use make it difficult to compare findings across studies [5, 12, 13]. To ensure reliable and valid estimates of PA, researchers must accurately quantify device wear time, enabling robust interpretation and meaningful comparisons between studies.

There is limited evidence on objectively measured PA in UK children with haemophilia across severities [14], and no studies have objectively measured PA or SB in BwH against WHO recommendations.

This study aimed to objectively assess PA and SB in UK BwH across severities. Secondary aims were to determine the proportion meeting WHO recommendations for daily MVPA, and to explore group characteristics associated with levels of PA and SB, for example, severity and BMI (Table S1).

## 2 Methods

Research ethics approval was provided by the Central London Research Ethics Committee (ref: 17/LO/1192) and the study is registered with clinicaltrials.gov (ID: NCT04076306).

This study includes detailed sub‐analysis of accelerometry data collected as part of the previously published primary study [14], the aim of which was to evaluate the feasibility, safety and acceptability of select outcome measures as part of a study protocol testing BwH; including myometry, 10 m incremental shuttle walk test (10‐m ISWT), iSTEP (an incremental step test) and 1 week of accelerometry‐wear at home.

Boys aged 6–15 years with all severities of haemophilia, including boys with an inhibitor who had been on emicizumab for 6 months, receiving care at Great Ormond Street Hospital for Children NHS Foundation Trust haemophilia centre (GOSH), were invited to participate in the study via letter and recruited between January and November 2018.

Age‐appropriate information sheets were provided and written parental consent and child assent were obtained. Participants who attended the GOSH clinic and consented during this period, provided accelerometry data. Participants were recruited sequentially and all eligible boys were approached during the study window.

Exclusion criteria included any bleed within the previous 4 weeks or the presence of co‐existing respiratory, cardiac, neurological or non‐haemophilia related orthopaedic conditions. No restrictions were applied with respect to treatment history.

Once consented, boys were screened before being fitted with an accelerometer, including specific questions about pain or symptoms of a bleed, on the day of screening and in the previous 4 weeks. If symptomatic, they were asked to identify the presence of pain on a body chart and give a score using the Wong‐Baker Faces Pain Rating Scale [15]. Reports of pain or bleeds were referred to the GOSH Haemophilia Physiotherapy Team who reviewed to confirm inclusion/exclusion. Families received education on accelerometer use, advised to maintain their normal PA levels and provided with a letter for schools explaining the study and the accelerometer‐wear protocol.

Demographic and clinical data extracted from medical notes included age, type and severity of haemophilia, treatment status, number of bleeds in the previous year, radiological evidence of arthropathy, height, weight and body mass index (BMI) and Haemophilia Joint Health Score (HJHS) [16].

Participants were asked to wear a calibrated ActiGraph GT3X bi‐axial accelerometer on the right hip for 7 days during waking hours, removing them for sleep and water‐based activities. They were returned in a stamped addressed envelope [17]. Actigraph accelerometers have been shown to provide valid estimates of SB and MVPA in children, but are less accurate for light activity, and results depend on device placement, algorithm and population calibration [18].

They were programmed to record dates of usage, to store data in 15‐s sampling epochs and to record activity as counts and steps. This is consistent with comparable accelerometry studies in BwH [13, 19, 20].

Data from each accelerometer were uploaded, and standard accelerometry outcomes were calculated using ActiGraph Firmware. Minimal wear time of 9 h a day (540 min) for 4 days was required for inclusion of each participants’ accelerometry data in the analysis [13, 21]. The thresholds defined by Evenson et al. [22] were used to calculate daily time spent in sedentary, light, moderate and vigorous activity and are recorded as counts per minute (CPM): Sedentary 0–100; Light 101 ≥ 2295; Moderate ≥ 2296; Vigorous ≥ 4012 [22]. All CPM ≥2296 were included for MVPA analysis.

### 2.1 Statistical Analysis

*Z*‐scores for BMI were calculated from the UK 1990 standards [23] and processed using the childsds R library [24]. Differences between disease severity groups were assessed with the Kruskal–Wallis (KW) test for continuous variables, and with the chi‐squared test for categorical variables. A two‐tailed significance level of *p *< 0.05 was used. All statistical models included a random effects term on their intercept to account for patients’ repeated measurements and were fitted using the lme4 R library [25]. We fitted linear mixed effects models to analyse the effects of clinical and demographic factors on the daily minutes spent in MVPA and SB, and mixed effects logistic regression models for the probability of achieving 60 min per day. Model selection was based on Akaike's Information Criterion (AIC) [26]. Conditional correlation coefficients for repeated measurements were obtained using the R library performance [27].

## 3 Results

Accelerometry data were collected between January and November 2018. Forty‐three boys were recruited to the study, and two accelerometers were not returned.

We report data from 36 boys who met the wear time validity of at least 9 h daily for at least 4 days. There were 216/252 (85.6%) potential days for analysis; 161 were weekdays and 55 weekend days. Sixteen boys had 7 valid days with 6, 5 and 4 valid days recorded by 9, 6 and 5 boys, respectively. Five boys did not meet wear time validity (>9 h, >4days) and were excluded. No bleeds were reported whilst wearing the accelerometer.

Demographic details of the 36 participants (median age 9.9 years (8.73, 11.55) are presented in Table 1, with 6 (16%) having mild/moderate disease, 23 (63%) severe and 7 (19%) having inhibitors. The majority 26/36 (72%) of children were receiving prophylaxis. All children with an inhibitor were on emicizumab for a minimum of 6 months. One child with an inhibitor had radiographic evidence of arthropathy at his knee. There were no significant associations between severity and age (KW test: *p* = 0.192), height in cm (KW test: *p* = 0.456) and zBMI (KW test: *p* = 0.440). HJHS score was zero in 12/36 (33%) children; 3 mild/moderate, 8 severe and 1 inhibitor. There was no significant difference in proportion of HJHS zero scores by disease severity (chi‐squared test, 2*df*: *p* = 0.384).

**TABLE 1:** Demographics and results.

	Mild/moderate (*n *= 6)	Severe (*n *= 23)	Inhibitor (*n *= 7)	Total (*n *= 36)
**Diagnosis** Haemophilia A Haemophilia B	3 (2 mild, 1 mod) 3 (3 mod)	18 5	7	28 8
Age (yrs)	9.15 (7.72, 11.77)	10.20 (9.25, 12.10)	8.80 (7.40, 10.15)	9.90 (8.73, 11.55)
**Treatment** Prophylaxis On demand Novel treatment^*^	3 (moderate) 3 (mild)	23	7	26 3 7
**Primary prophylaxis (<2 years of age)** **Secondary prophylaxis (>2 years of age)**		20 3	6 1	26 4
**HJHS** ^+^ Median/IQR	0.67 (0‐2)	2.57 (0‐12)	3 (0‐8)	2.52 (0‐12)
**zBMI** **Median/ IQR**	0.78 (‐1.7‐2.4)	0.11 (‐2.4‐ 2.5)	0.24 (‐1.9‐2.4)	0.24 (‐2.47‐2.5)
**Height (cm)**	135.48 (115.0‐156.4)	142.34 (120.7‐167.7)	134.90 (113.9‐150.0)	139.8 (113.9‐167.7)
**MVPA per day in minutes Median / IQR)**	45.8 (20.8, 71.3)	55.4 (30.9, 79.6)	54.3 (38.8, 86.1)	52.5 (30.9‐80.2)
**Sedentary behaviour per day in minutes** **Median / IQR)**	469.8 (394.3, 547.8)	483.8 (404.05, 573.4)	436.6 (350.1,497.2)	476 (391.4‐557.8)
**Step count** ^**^ Median/IQR	9100 (7051, 12370)	9002 (7525, 12084)	11148 (9386,14005)	9754 (7441,13088)

* Novel treatment involves inhibitor management with emicizumab‐ only children with an inhibitor.

^+^ HJHS included the global gait score.

** Median step count and IQR for the valid days only.

### 3.1 Moderate to Vigorous PA

There was no difference in MVPA between weekdays versus weekends (*p* = 0.096). There was no significant difference between severity groups for average MVPA per day in minutes (KW test: *p* = 0.6578). Age was associated with MVPA for all groups; with a decline in daily MVPA observed with increased age (*p* < 0.01) (see Figure 1). On average, with every 1‐year increase in age BwH lose 7.62 min of MVPA yielding a loss of 38 min of average daily MVPA in 5 years. Correlation coefficients between age and MVPA adjusted for repeated measurements were −0.713, −0.765 and −0.498 for Severe, Mild/Moderate and Inhibitor groups respectively, all significant at *p* < 0.01.

**FIGURE 1:**
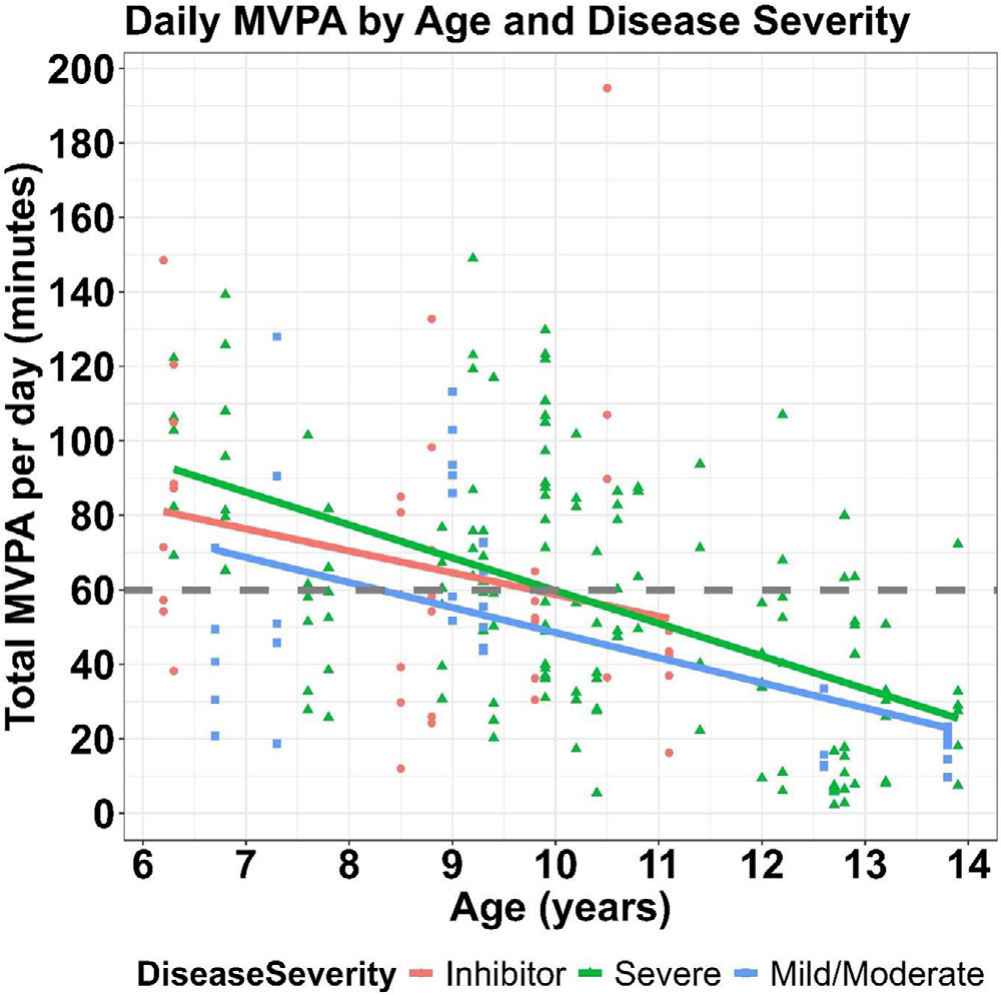
Daily MVPA by disease severity and age. Each point represents daily total MVPA minutes completed for each child. The range of daily totals for each child is displayed vertically. The colour/shape reflect disease severity. The solid line is the best fit line for each severity from the mixed‐effects model.

### 3.2 Sedentary Behaviour

SB in minutes did not differ by weekday versus weekend (*p* = 0.117) or by severity (KW *t*: *p* = 0.052). Age influenced SB for all groups (*p *< 0.01); with an increase in daily SB observed with increased age (Figure 2). On average for every 1‐year increase in age BwH increased their SB by 27.56 min equating to 2 h 18 min of extra sedentary time in 5 years.

**FIGURE 2:**
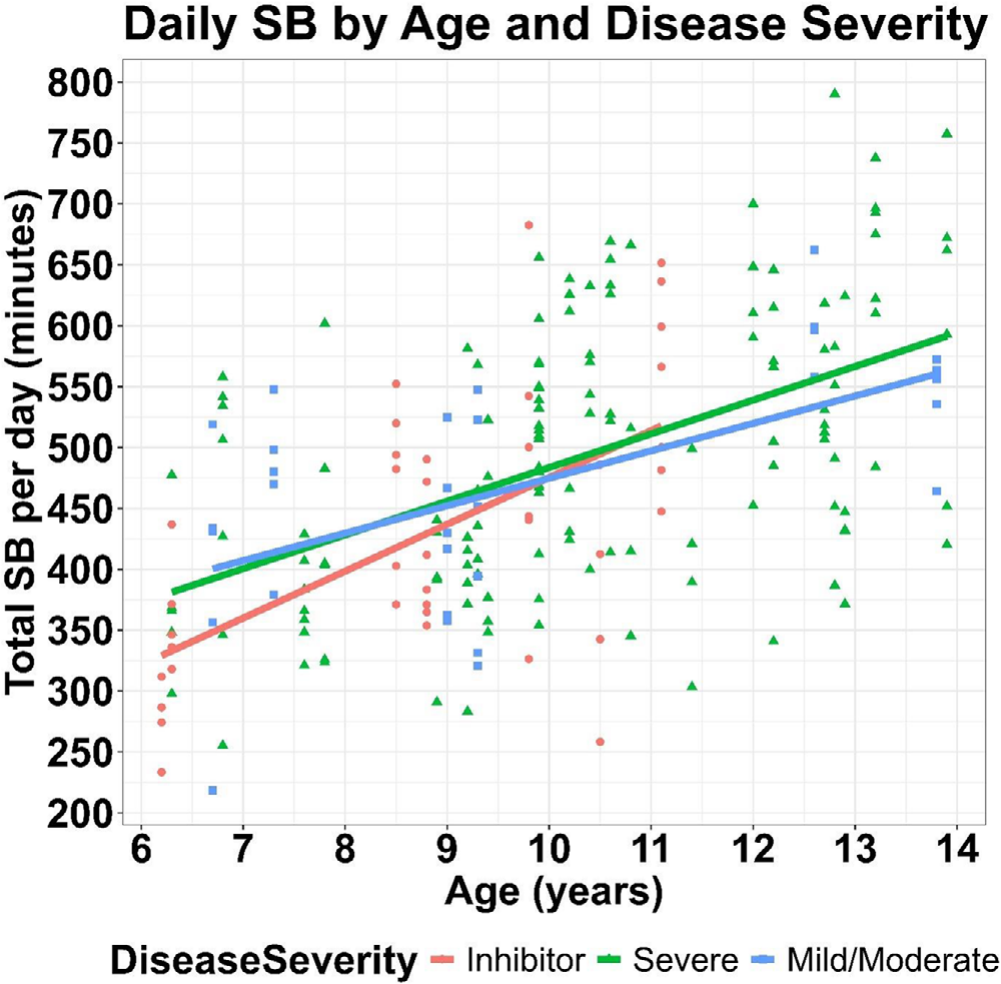
Daily SB by age and disease severity. Each point represents daily total of sedentary behaviour in minutes for each child. The range of daily totals for each child is displayed vertically. The colour/shape reflect disease severity. The solid line is the best fit line for each severity from the mixed‐effects model.

Correlation coefficients between age and SB adjusted for repeated measurements were 0.589, 0.692 and 0.733 for Severe, Mild/Moderate and Inhibitor groups, respectively, all significant at *p *< 0.01. There were no significant differences between the trajectories for the three disease severity groups.

### 3.3 60‐Min WHO Recommendation

Across 216 days, there were 86 days (39.8%) which met or surpassed the WHO daily 60 min MVPA recommendation, but 130 days (60.2%) which did not.

Only age was a predictor of achieving the 60‐min daily MVPA (*p* < 0.001), neither HJHS, disease severity nor zBMI were significant (*p *> 0.05). The odds of completing >60 min/day MVPA decreased by 37.5% per year of age (age effect: odds ratio estimate 0.63, 95% confidence interval 0.49–0.80, *p *< 0.01). The probability of a 6‐year‐old BwH achieving the 60‐min daily MVPA recommendation was 79%, declining to 8% at age 14. At 8.75 years the probability of reaching > 60 min MVPA is 50% (Figure 3). Half of the boys are predicted to complete 60 min a day at 9 years of age.

**FIGURE 3:**
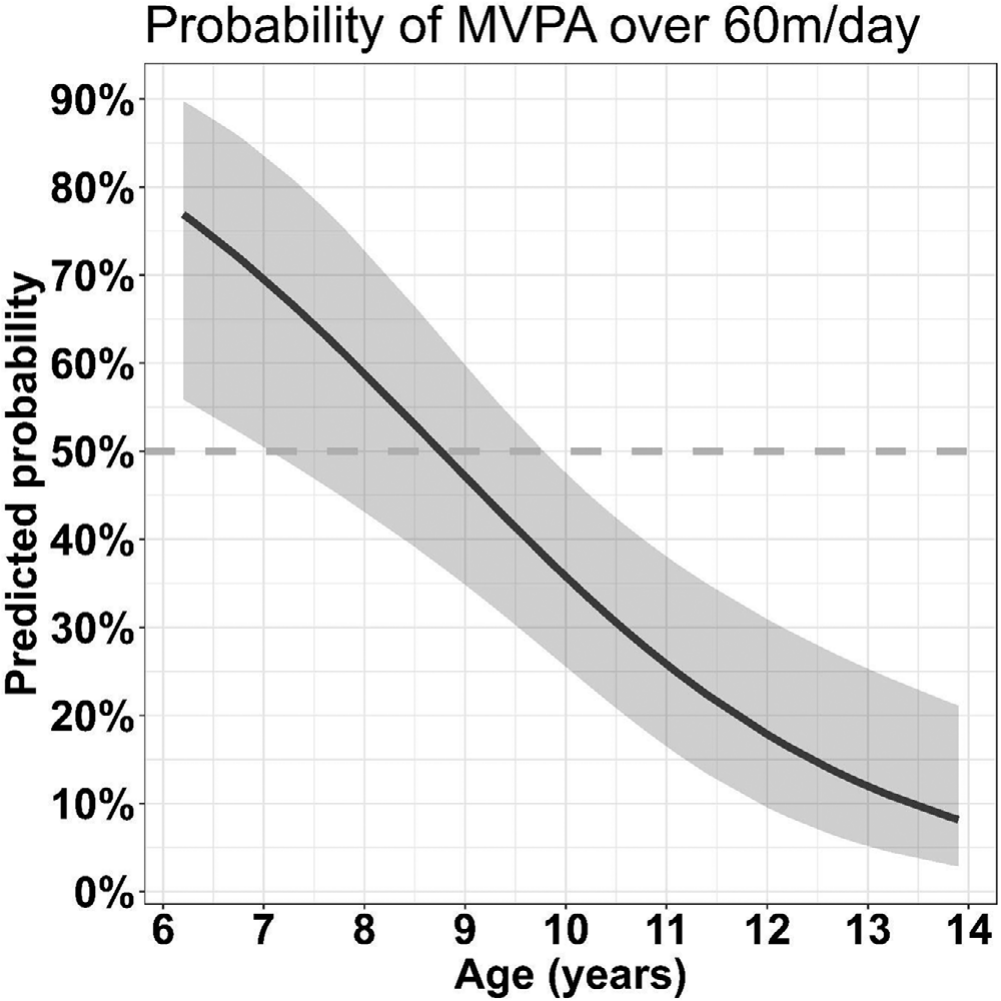
Probability of MVPA over 60m/day. As age increases the probability of an individual BwH completing 60m of MVPA in a day reduces. Half of boys are predicted to complete 60 min a day at 9 years of age.

There was no significant difference between disease severity and step counts per valid day. (KW test: *p* = 0.5222). The average step count for BwH was 9754 (7441,13088); with boys with an inhibitor achieving the most steps per valid day 11148 (9386, 14005) (see Table 1).

## 4 Discussion

This is the first UK accelerometry study to assess PA and SB in children with haemophilia across all severities including inhibitors, benchmarked against WHO guidelines for MVPA [4]. BwH have relatively high MVPA levels in comparison to their peers but fall short of the WHO guidelines with a strong age effect. To ensure reliable estimates, we included only data from boys meeting wear‐time validity criteria, enabling accurate interpretation of MVPA and sedentary time and meaningful comparisons with studies using similar protocols.

Using Evenson thresholds, we found that most BwH at our centre did not meet the WHO‐recommended 60 min of daily MVPA, averaging 52.5 min (IQR: 30.9–80.2). In the general population, 81% of adolescents aged 11–17 years do not meet WHO MVPA guidelines [28], and only 47% of children aged 5–16 in England meet them [29]. Our findings align with previous literature showing no association between disease severity and MVPA, despite differences in age, time‐period and variation in treatment.

While a 2021 systematic review [5] reported increased PA in PwH over recent years, our cohort showed substantial individual variability in MVPA and sedentary time. Few accelerometry studies in children with haemophilia are available for direct comparison, and most are over a decade old [13, 19, 20]. Buxbaum [19] (2010; mean age 13.7) and Bouskill [13] (2016; mean age 11.7) also reported no significant MVPA differences by severity, consistent with our results (median ages: 9.15, 7.12, 11.77).

In Gonzalez's 2010 study [20] (mean age 12.8), BwH demonstrated higher light, moderate and MVPA levels than controls (*p* < 0.001), yet neither group exceeded 9 min of MVPA daily—substantially lower than in our cohort. This may reflect improvements in PA over time, though differences in MVPA calculation methods warrant further exploration. Advances in haemophilia management since then likely contribute to increased activity levels.

We observed an age‐related decline in MVPA, consistent with Bouskill's findings, despite differences in age ranges. An MVPA decline with age is well‐documented in the general population. A meta‐analysis of accelerometry studies [30] identified a notable drop in MVPA around age 9 in boys, which mirrors the trend in our cohort, though our study was limited in power. Factors positively associated with sustained PA include male gender, perceived competence and prior PA exposure [31]. Our data showed a decreasing likelihood of meeting the 60‐min MVPA target with age. Despite medical advances, PA in BwH appears to decline similarly to their unaffected peers, underscoring the need for early, proactive strategies to promote and maintain activity, for example family values and physical literacy.

While previous studies [13, 19, 20] reported increased SB in boys with severe haemophilia, we found no such difference—possibly reflecting improved medical management. The HJHS in this study were low across all groups, including severe forms of the disease, reflecting minimal or no clinically detectable joint damage. It could be hypothesised that joint health is not restricting MVPA or contributing to SB in this cohort. Further exploration of the determinants of PA and SB should be explored, for example parental overprotection, habitual patterns, lifestyle and physical literacy. Alternatively, the sensitivity of the HJHS with a significant floor effect may be contributing to weaker correlations between joint health and PA/SB.

Methodological differences and the absence of HJHS in earlier studies limit direct comparisons. Bouskill [13] suggested that increased SB in severe cases may relate to arthropathy or psychological factors. In our cohort, SB increased with age across all severities (*p* < 0.01), consistent with trends in the general population [32]. SB, while part of the movement spectrum, can negatively impact health and is shaped by social, environmental, economic and technological factors [3]. Post the COVID pandemic, PA levels in children have largely returned to normal, but SB has increased, likely due to a shift toward structured activities and reduced opportunities for spontaneous play. This suggests that the SB observed in 2018 may be further increased in 2026. Future interventions should prioritise reducing SB, not just maintaining PA.

Although BwH are now more active [5], they still fall short of WHO MVPA recommendations. Our findings align with previous studies [12, 13] despite differences in methodology and devices used. Bouskill et al. [13] reported average MVPA of 48 ± 20 min (severe) and 55 ± 18 min (mild/moderate), similar to our results. Notably, in our cohort, boys with severe disease or inhibitors had MVPA levels closer to the 60‐min target—averaging 55.4 and 54.3 min respectively—while the mild/moderate group averaged lower at 45.8 min.

Matlary [12] reported a case–control study of Norwegian teenagers with haemophilia (mean age 19.5) using Fitbit devices over 12 weeks. Only 40% (*n* = 8) met the weekly 420 min MVPA target, compared to 8% of controls. Direct comparisons with our study are limited due to differences in devices, severity, age groups and definitions of valid data (e.g., >1000 steps/day). Despite methodological variation, a consistent theme emerges: most adolescents with haemophilia do not meet WHO MVPA recommendations.

This is the first study to quantify PA, SB and WHO guideline adherence in UK BwH with an inhibitor receiving a bispecific monoclonal antibody (median age 8.8). Inhibitor presence remains a significant challenge in haemophilia care, often linked to poorer quality of life. Nonetheless, PA is encouraged for this group, and interim data suggest it is safe [33]. However, literature on PA in children with inhibitors remains limited [7, 14]. Encouragingly in our study, these children engaged in MVPA without reported bleeds during monitoring. While disease severity did not predict MVPA adherence, the specific types of PA in the inhibitor group remain unknown and warrant further study.

The apparent group difference in MVPA may simply be an age affect, as the mild/ moderate group included more older boys, but may also reflect lifestyle preferences related to reactive rather than preventative treatment. There are alternative, plausible factors.

Transition to effective prophylaxis for those with an inhibitor may evidence more stable haemostatic protection, lower bleed rates compared to previous and reduced treatment burden. As a result of these changes, they may now feel safer, more confident engaging in physical play and sports, have a desire to catch up or parental willingness to relax restrictions. Paradoxically, this may contrast to those parents of children with mild or moderate disease who may be more uncertain about activity risk because their children bleed less predictably. For those children with severe haemophilia on prophylaxis breakthrough bleeds may still occur due to trauma, adherence challenges and variable minimum trough levels. These factors may contribute to children keeping their MVPA lower when prophylaxis is nominally effective. These findings may contrast significantly to the older generation of PwH who were discouraged from engaging in PA and where treatment was limited.

Interestingly, children with an inhibitor recorded the highest step count per valid day (median 11,148; IQR 9386–14,005), though not the highest MVPA (54.3 min; IQR 38.8–86.1). While step count has been reported in PwH, data specific to children are lacking [34]. The relationship between steps and MVPA is inconsistent, with adult studies suggesting 7000–8000 steps may equate to 30 min of MVPA [35]. However, WHO guidelines note insufficient evidence linking step count to health outcomes [36]. In our study, MVPA and step count varied independently, likely influenced by factors such as age, sex, cadence and intensity [37]. Both metrics are important for assessing adherence to health recommendations.

Tudor‐Locke et al. [35] recommend 13,000–15,000 steps/day for school‐aged boys. In the UK Millennium Cohort Study, 7–8‐year‐old boys averaged 10,739 steps/day, with 63% meeting WHO MVPA guidelines [38]. In our cohort (median age 9.9), BwH averaged fewer steps—9754/day (IQR 7441–13,088)—yet 70% of 7‐year‐olds were predicted to meet the MVPA target. Despite differences in age, methodology and post‐pandemic context, our findings suggest that BwH are approaching recommended activity levels of MVPA and step count. This is encouraging and reflects the positive impact of modern haemophilia care and proactive clinical support for PA. PA choices for BwH should be individualised, with shared decision‐making between families and the multidisciplinary team [34]. In our study, age was the only significant predictor of MVPA, though broader determinants—individual, social, environmental and policy‐related—also influence activity levels.

Perceived competence, a key component of physical literacy, may also play a role. Research suggests that psychological factors, rather than motor or fitness deficits, are more influential in PA participation among children with disabilities [1, 39]. Future studies should explore the role of physical literacy in BwH.

Looking ahead, the global rise in obesity—projected to affect 60% of adults and 31% of children by 2050 [40] poses additional risks for those with chronic conditions. Although no group differences in zBMI were observed in our study, and no children were obese, the trend is concerning. PA is protective against obesity, reinforcing the importance of promoting activity and reducing SB from early childhood [28, 41].

### 4.1 Limitations

zBMI was not a predictor of achieving 60‐min daily MVPA. BMI does have limitations [42], but we had no other measures of adiposity. Other measures of obesity are relevant and should be explored as clinicians continue to promote wellbeing.

In 2026, PA continues to be advocated for children with haemophilia, and clinicians emphasise the need for objective PA data to guide counselling and prophylaxis optimisation. We acknowledge that the data was collected in 2018, but there remains a striking lack of contemporary research grade PA data in BwH. Most publications available are either self‐reported, evaluate adults, focus on treatment outcomes or concentrate on self‐reports of sports participation rather than direct measurement. Studies that describe PA in a mixed cohort of BwH across all severities and with detailed and reliable activity intensity data are exceptionally rare. This study is one of the largest and only objective PA data sets on children with haemophilia reported internationally.

The impact of widespread adoption of emicizumab could have influenced confidence for boys to participate in activity and for their family and care team to encourage it. 2018 was a transitional period when some but not all children were eligible for emicizumab in the UK. This study offers objective PA data before universal adoption as only those with an inhibitor were on emicizumab. This study provides a critical baseline for evaluating changes in PA over time. PA levels have changed free living PA patterns, especially for those with an inhibitor. Current literature still lacks robust evidence demonstrating how the introduction of novel agents has altered PA patterns. This data provides important historical and clinical utility value and will permit meaningful comparisons against current and future landscapes. Contextualisation strengthens the manuscripts contribution to PA in BwH in an evolving landscape.

Variability in how MVPA and SB are calculated across devices complicates comparisons between studies. Differences in device setup, cut‐points and methodology—particularly between hip‐ and wrist‐worn monitors—limit consistency. The ActiGraph, while widely used, is not a criterion measure and may miss non‐ambulatory activities (e.g., carrying loads, swimming). Although hip‐worn devices often have lower compliance, our participants averaged six valid wear days and patient satisfaction with use over this duration has been supported [43]. Actigraphs do not provide feedback like many consumer‐purchased devices. For longer‐term monitoring, wrist‐worn multi‐sensor trackers (e.g., Fitbit) may offer better compliance and estimates for diverse activity types [44].

However, proprietary algorithms based on adult heart rate data and inconsistent definitions of valid days further hinder comparability. A recent meta‐analysis found that wearables may increase step counts in children, perhaps related to live feedback, but not MVPA highlighting the importance of study design and device selection in PA research [45]. Seasonality is an important consideration that we were unable to explore and should be considered in future studies [46].

## 5 Conclusion

Advances in haemophilia care have significantly reduced bleeding frequency, enabling most children on prophylaxis to lead active lives. Most BwH in our study did not meet WHO MVPA guidelines, but they came close—highlighting encouraging progress in clinical management.

Age‐related decline in MVPA remains a concern and increasing sedentary time problematic especially for an ageing population of PwH who may have increased cardiometabolic risk factors. Interventions should begin in childhood with the aim to understand family values around PA, tailor safe activity choices and prevent this decline, focusing on understanding and addressing the root causes of inactivity.

## Author Contributions

**Melanie Bladen**: Chief Investigator, designed the original study, wrote the manuscript and contributed to data interpretation. **Lucy Alderson**: contributed to data interpretation and revisions of the manuscript. **Eleanor Main**: contributed to data interpretation. **Mario Cortina‐Borja**: analysed the data and contributed to data interpretation. All authors contributed to writing the manuscript. All authors approved the final manuscript.

## Ethics Statement

Research Ethics Approval was provided by the Central London Research Ethics Committee (ref: 17/LO/1192) 2017. The study was conducted in line with the International Council for Harmonisation E6 guideline for Good Clinical Practice and the principles of the Declaration of Helsinki.

## Consent

All participants provided written consent or assent for participation.

## Conflicts of Interest

Melanie Bladen has served on an advisory board to CSL, has received research support from Pfizer, Sobi and Roche. The remaining authors have no competing interests.

## Supporting information




**Supporting Table 1**: Summary statistics for 43 children recruited in the study; the accelerometry variables refer to 41 children who returned the accelerometers. **Supporting Table 2**: Coefficients from the mixed‐effects model for total MVPA per day (minutes); the reference category for Disease severity is Inhibitor. **Supporting Table 3**: Coefficients from the mixed‐effects model for SB per day (minutes); the reference category for Disease severity is inhibitor.

## Data Availability

The data that support the findings of this study are available from the corresponding author upon reasonable request.

## References

[1] V. J. Poitras , C. E. Gray , M. M. Borghese , et al., “Systematic Review of the Relationships Between Objectively Measured Physical Activity and Health Indicators in School‐Aged Children and Youth,” Applied Physiology, Nutrition, and Metabolism 41, no. 6 Suppl 3 (2016): S197–S239, 10.1139/apnm-2015-0663. 27306431

[2] H. W. Kohl , C. L. Craig , E. V. Lambert , et al., “The Pandemic of Physical Inactivity: Global Action for Public Health,” Lancet 380 (2012): 294–305. 10.1016/S0140-6736(12)60898-8 22818941

[3] A. J. Pinto , A. Bergouignan , P. C. Dempsey , et al., “Physiology of Sedentary Behavior,” Physiological Reviews 103, no. 4 (2023): 2561–2622, 10.1152/physrev.00022.2022. PMC10625842 37326297

[4] World Health Organization Guidelines on Physical Activity and Sedentary Behaviour Geneva, Switzerland 2020. 33369898

[5] M. Kennedy , P. O'Gorman , A. Monaghan , et al., “A Systematic Review of Physical Activity in People With Haemophilia and Its Relationship With Bleeding Phenotype and Treatment Regimen,” Haemophilia 27, no. 4 (2021): 544–562, 10.1111/hae.14282. PMC8359343 33751742

[6] T. Hereford , A. Kellish , J. B. Samora , and L. Reid Nichols , “Understanding the Importance of Peak Bone Mass,” Journal of the Pediatric Orthopaedic Society of North America 7 (2024): 100031, 10.1016/j.jposna.2024.100031. PMC12088333 40433296

[7] A. Srivastava , E. Santagostino , A. Dougall , et al., “WFH Guidelines for the Management of Hemophilia, 3rd Edition,” Haemophilia. 26, no. S6 (2020): 1–158, 10.1111/hae.14046. 32744769

[8] M. Kennedy , S. Roche , M. McGowan , et al., “Physical Activity, Physical Fitness and Cardiometabolic Risk amongst Adults With Moderate and Severe Haemophilia,” Haemophilia 29, no. 1 (2022): 72–83, 10.1111/hae.14653. PMC10092720 36195106

[9] S. Kwon , K. F. Janz , E. M. Letuchy , T. L. Burns , and S. M. Levy , “Developmental Trajectories of Physical Activity, Sports, and Television Viewing during Childhood to Young Adulthood,” JAMA Pediatrics 169, no. 7 (2015): 666, 10.1001/jamapediatrics.2015.0327. PMC4596396 25984811

[10] R. Telama , X. Yang , E. Leskinen , et al., “Tracking of Physical Activity From Early Childhood Through Youth Into Adulthood,” Medicine & Science in Sports & Exercise 46, no. 5 (2014): 955–962, 10.1249/MSS.0000000000000181. 24121247

[11] M. A. Timmer , S. C. Gouw , B. M. Feldman , et al., “Measuring Activities and Participation in Persons With Haemophilia: A Systematic Review of Commonly Used Instruments,” Haemophilia 24, no. 2 (2018): e33–e49. 10.1111/hae.13367 29178149

[12] R. E. D. Matlary , M. Grydeland , H. Glosli , C. S. Rueegg , and P. A. Holme , “Physical Activity in Norwegian Teenagers and Young Adults With Haemophilia A Compared to General Population Peers,” Haemophilia 29, no. 2 (2023): 658–667, 10.1111/hae.14752. 36723510

[13] V. Bouskill , P. Hilliard , S. Stephens , C. Zhang , K. Whitney , and M. Carcao , “An Institutional Pilot Study to Investigate Physical Activity Patterns in Boys With Haemophilia,” Haemophilia 22, no. 5 (2016): e383–e389. 10.1111/hae.13021 27530715

[14] M. Bladen , L. Alderson , N. Thorpe , M. Cortina‐Borja , and E. Main , “Performance on the iSTEP and 10 M‐ISWT in Boys With Haemophilia,” Haemophilia 29, no. 5 (2023): 1343–1350, 10.1111/hae.14833. 37572336

[15] J. F. Keck , J. E. Gerkensmeyer , B. A. Joyce , and J. G. Schade , “Reliability and Validity of the Faces and Word Descriptor Scales to Measure Procedural Pain,” Journal of Pediatric Nursing 11, no. 6 (1996): 368–374. 10.1016/S0882-5963(96)80081-9 8991337

[16] B. M. Feldman , S. M. Funk , B.‐M. Bergstrom , et al., “Validation of a New Pediatric Joint Scoring System From the International Hemophilia Prophylaxis Study Group: Validity of the Hemophilia Joint Health Score,” Arthritis Care & Research 63, no. 2 (2011): 223–230, 10.1002/acr.20353. 20862683

[17] S. M. Phillips , C. Summerbell , M. Hobbs , et al., “A Systematic Review of the Validity, Reliability, and Feasibility of Measurement Tools Used to Assess the Physical Activity and Sedentary Behaviour of Pre‐school Aged Children,” International Journal of Behavioral Nutrition and Physical Activity 18 (2021): 141, 10.1186/s12966-021-01132-9. PMC8567581 34732219

[18] R. M. Pulsford , M. Cortina‐Borja , C. Rich , F. E. Kinnafick , C. Dezateux , and L. J. Griffiths , “Actigraph Accelerometer‐Defined Boundaries for Sedentary Behaviour and Physical Activity Intensities in 7 Year Old Children,” PLoS ONE 6, no. 8 (2011): e21822, 10.1371/journal.pone.0021822. PMC3154898 21853021

[19] N. P. Buxbaum , M. Ponce , P. Saidi , and L. A. Michaels , “Psychosocial Correlates of Physical Activity in Adolescents With Haemophilia,” Haemophilia 16 (2010): 656–661. 10.1111/j.1365-2516.2010.02217.x 20331760

[20] L. M. González , C. Peiró‐Velert , J. Devís‐Devís , et al., “Comparison of Physical Activity and Sedentary Behaviours Between Young Haemophilia A Patients and Healthy Adolescents,” Haemophilia 17, no. 4 (2011): 676–682. 10.1111/j.1365-2516.2010.02469.x 21299746

[21] C. Rich , M. Geraci , L. Griffiths , F. Sera , C. Dezateux , and M. Cortina‐Borja , “Quality Control Methods in Accelerometer Data Processing: Defining Minimum Wear Time,” PLoS ONE 8 (2013): e67206, 10.1371/journal.pone.0067206. PMC3691227 23826236

[22] K. R. Evenson , D. J. Catellier , K. Gill , K. S. Ondrak , and R. G. McMurray , “Calibration of Two Objective Measures of Physical Activity for Children,” Journal of Sports Sciences 26, no. 14 (2008): 1557–1565, 10.1080/02640410802334196. 18949660

[23] T. J. Cole , J. V. Freeman , and M. A. Preece , “British 1990 Growth Reference Centiles for Weight, Height, Body Mass Index and Head Circumference Fitted by Maximum Penalized Likelihood,” Statistics in Medicine 17, no. 4 (1998): 407–429. 9496720

[24] M. Vogel , _childsds: Data and Methods Around Reference Values in Pediatrics, R package version 0.9.8 (2025), https://CRAN.R‐project.org/package=childsds.

[25] D. Bates , M. Mächler , B. Bolker , and S. Walker , “Fitting Linear Mixed‐Effects Models Using lme4,” Journal of Statistical Software 67, no. 1 (2015): 1–48, 10.18637/jss.v067.i01.

[26] J. Kuha , “AIC and BIC,” Sociological Methods & Research 33, no. 2 (2004): 188–229, 10.1177/0049124103262065.

[27] D. Lüdecke , M. S. Ben‐Shachar , I. Patil , P. Waggoner , and D. Makowski , “performance: An R Package for Assessment, Comparison and Testing of Statistical Models,” Journal of Open Source Software 6, no. 60 (2021): 3139, 10.21105/joss.03139.

[28] R. Guthold , G. A. Stevens , L. M. Riley , and F. C. Bull , “Global Trends in Insufficient Physical Activity Among Adolescents: A Pooled Analysis of 298 Population‐Based Surveys With 1·6 Million Participants,” Lancet Child Adolesc Health 4, no. 1 (2020): 23–35, 10.1016/S2352-4642(19)30323-2. PMC6919336 31761562

[29] Sport England , “Active Lives Children and Young People Survey Academic year 2022‐23,” Available at: Active Lives Children and Young People Survey—Academic year 2022–23 (Accessed 14.01.2025) (2023a).

[30] A. Farooq , A. Martin , X. Janssen , et al., “Longitudinal Changes in Moderate‐to‐Vigorous‐Intensity Physical Activity in Children and Adolescents: A Systematic Review and Meta‐Analysis,” Obesity Reviews 21, no. 1 (2019): e12953, 10.1111/obr.12953. PMC6916562 31646739

[31] A. E. Bauman , R. S. Reis , J. F. Sallis , J. C. Wells , R. J. Loos , and B. W. Martin , “Correlates of Physical Activity: Why Are some People Physically Active and Others Not?,” The Lancet 380, no. 9838 (2012): 258–271, 10.1016/S0140-6736(12)60735-1. 22818938

[32] E. Kontostoli , A. P. Jones , N. Pearson , L. Foley , S. J. H. Biddle , and A. J. Atkin , “Age‐Related Change in Sedentary Behavior During Childhood and Adolescence: A Systematic Review and Meta‐Analysis,” Obesity Reviews 22, no. 9 (2021): e13263, 10.1111/obr.13263. 34080284

[33] J. Mahlangu , J. Oldenburg , M. U. Callaghan , et al., “Health‐Related Quality of Life and Health Status in Persons With Haemophilia A With Inhibitors: a Prospective, Multicentre, Non‐Interventional Study (NIS),” Haemophilia 25, no. 3 (2019): 382–391, 10.1111/hae.13731. PMC6850115 31016855

[34] R. E. D. Matlary , N. Grinda , F. Sayers , O. Versloot , and P. McLaughlin , “Promoting Physical Activity for People With Haemophilia in the Age of New Treatments,” Haemophilia 28, no. 6 (2022): 885–890, 10.1111/hae.14641. PMC9796679 35896000

[35] C. Tudor‐Locke , C. Leonardi , W. D. Johnson , P. T. Katzmarzyk , and T. S. Church , “Accelerometer Steps/Day Translation of Moderate‐to‐Vigorous Activity,” Preventive Medicine 53, no. 1–2 (2011): 31–33, 10.1016/j.ypmed.2011.01.014. 21295063

[36] J. L. Mair , E. J. Aguiar , E. Stamatakis , and S. M. Edney , “Moving Toward the Inclusion of Step‐Based Metrics in Physical Activity Guidelines and Surveillance,” Journal of Physical Activity and Health 20, no. 7 (2023): 575–577, 10.1123/jpah.2023-0180. 37279896

[37] C. D. Patnode , L. A. Lytle , D. J. Erickson , J. R. Sirard , D. Barr‐Anderson , and M. Story , “The Relative Influence of Demographic, Individual, Social, and Environmental Factors on Physical Activity Among Boys and Girls,” International Journal of Behavioral Nutrition and Physical Activity 7 (2010): 79, 10.1186/1479-5868-7-79. PMC2991277 21047429

[38] L. J. Griffiths , M. Cortina‐Borja , F. Sera , et al., “How Active Are Our Children? Findings From the Millennium Cohort Study,” BMJ Open 3, no. 8 (2013): e002893, 10.1136/bmjopen-2013-002893. PMC3752053 23965931

[39] P. E. Longmuir , A. Chubbs Payne , N. Beshara , et al., “Quick, Effective Screening Tasks Identify Children with Medical Conditions or Disabilities Needing Physical Literacy Support,” Pediatric Exercise Science 37, no. 1 (2024): 18–28, 10.1123/pes.2023-0130. 38171358

[40] GBD 2021 Adult BMI Collaborators , “Global, Regional, and National Prevalence of Adult Overweight and Obesity, 1990–2021, With Forecasts to 2050: A Forecasting Study for the Global Burden of Disease Study 2021,” The Lancet 405, no. 10481 (2025): 813–838, 10.1016/S0140-6736(25)00355-1. PMC11920007 40049186

[41] Sport England , “Children's Physical Activity Levels Hold Firm but Significant Challenges Remain,” Available at: Children's activity levels hold firm but significant challenges remain | Sport England (Accessed 14.01.2025) (2023b).

[42] Y. Wu , D. Li , and S. H. Vermund , “Advantages and Limitations of the Body Mass Index (BMI) to Assess Adult Obesity,” International Journal of Environmental Research and Public Health 21, no. 6 (2024): 757, 10.3390/ijerph21060757. PMC11204233 38929003

[43] N. Thorpe , P. Harniess , E. Main , et al., “Feasibility, Safety and Acceptability of Select Outcome Measures in a Physiotherapy Study Protocol for Boys With Haemophilia,” Pilot Feasibility Studies 7, no. 1 (2021): 105, 10.1186/s40814-021-00831-1. PMC8099992 33957997

[44] R. P. Troiano , E. Stamatakis , and F. C. Bull , “How Can Global Physical Activity Surveillance Adapt to Evolving Physical Activity Guidelines? Needs, Challenges and Future Directions,” British Journal of Sports Medicine 54, no. 24 (2020): 1468–1473. 10.1136/bjsports-2020-102621 PMC7719905 33239352

[45] W. W. Au , F. Recchia , D. Y. Fong , et al., “Effect of Wearable Activity Trackers on Physical Activity in Children and Adolescents: A Systematic Review and Meta‐Analysis,” The Lancet Digital Health 6, no. 9: e625–e639. 10.1016/S2589-7500(24)00139-0 39112110

[46] A. Garriga , N. Sempere‐Rubio , M. J. Molina‐Prados , and R. Faubel , “Impact of Seasonality on Physical Activity: A Systematic Review,” International Journal of Environmental Research and Public Health 19, no. 1 (2021): 2, 10.3390/ijerph19010002. PMC8751121 35010262

