# Supplementary material for: Physical Activity and Sedentary Behaviour in Children With Haemophilia
Source: Haemophilia. 2026 Jul 3;32(6):1505–12. doi: 10.1111/hae.70347 (PMC13754631; doi:10.1111/hae.70347)
Supplement: Supplementary file 1 [file HAE-32-1505-s001.docx]

Supplementary

Table S1: Summary statistics for 43 children recruited in the study; the accelerometry variables refer to 41 children who returned the accelerometers.

|  | min | mean | max |
| --- | --- | --- | --- |
| Age (years) | 6.0 | 9.8 | 12.0 |
| Total HJHS | 0 | 2.2 | 12 |
| MVPA per day (minutes) | 7.5 | 51.5 | 105.1 |
| Sedentary behaviour per day (minutes) | 147.5 | 437.8 | 658.9 |
| Step counts per day | 2645 | 8306 | 14224 |

Table S2 Coefficients from the mixed-effects model for total MVPA per day (minutes); the reference category for Disease severity is Inhibitor

|  | Estimate | Standard Error | *t-*value | *p*-value |
| --- | --- | --- | --- | --- |
| Intercept | 135.74 | 16.99 | 7.99 | <0.01 |
| Age | -7.62 | 1.59 | -4.78 | <0.01 |
| Disease severity mild/mod | -12.89 | 9.26 | -1.39 | 0.17 |
| Disease severity severe | 0.96 | 9.02 | 0.11 | 0.92 |

Table S3 – Coefficients from the mixed-effects model for SB per day (minutes); the reference category for Disease severity is inhibitor.

|  | Estimate | Standard Error | *t-*value | *p*-value |
| --- | --- | --- | --- | --- |
| Intercept | 182.84 | 46.62 | 3.92 | < 0.01 |
| Age | 27.56 | 4.64 | 5.93 | <0.01 |
| Disease severity mild/mod | 26.18 | 26.31 | 1.00 | 0.33 |
| Disease severity severe | 20.92 | 33.15 | 0.63 | 0.53 |
